# Combining Cellular Immunization and Phage Display Screening Results in Novel, FcγRI-Specific Antibodies

**DOI:** 10.3390/v16040596

**Published:** 2024-04-12

**Authors:** Steffen Krohn, Tosca Holtrop, Arianne M. Brandsma, Petra Moerer, Maaike Nederend, Nikos Darzentas, Monika Brüggemann, Katja Klausz, Jeanette H. W. Leusen, Matthias Peipp

**Affiliations:** 1 Division of Antibody-Based Immunotherapy, Department of Internal Medicine II, University Medical Center Schleswig-Holstein and Christian-Albrechts-University Kiel, 24105 Kiel, Germany; 2 Center for Translational Immunology, University Medical Center Utrecht, Heidelberglaan 100, 3584 CX Utrecht, The Netherlandsj.h.w.leusen@umcutrecht.nl (J.H.W.L.); 3 Unit for Hematological Diagnostics, Department of Internal Medicine II, University Medical Center Schleswig-Holstein and Christian-Albrechts-University Kiel, 24105 Kiel, Germany

**Keywords:** Fc receptor, FcγRI, phage display, cellular panning, NGS, ELISA

## Abstract

Antibodies that specifically bind to individual human fragment crystallizable γ receptors (FcγRs) are of interest as research tools in studying immune cell functions, as well as components in bispecific antibodies for immune cell engagement in cancer therapy. Monoclonal antibodies for human low-affinity FcγRs have been successfully generated by hybridoma technology and are widely used in pre-clinical research. However, the generation of monoclonal antibodies by hybridoma technology that specifically bind to the high-affinity receptor FcγRI is challenging. Monomeric mouse IgG2a, IgG2b, and IgG3 bind human FcγRI with high affinity via the Fc part, leading to an Fc-mediated rather than a fragment for antigen binding (Fab)-mediated selection of monoclonal antibodies. Blocking the Fc-binding site of FcγRI with an excess of human IgG or Fc during screening decreases the risk of Fc-mediated interactions but can also block the potential epitopes of new antibody candidates. Therefore, we replaced hybridoma technology with phage display of a single-chain fragment variable (scFv) antibody library that was generated from mice immunized with FcγRI-positive cells and screened it with a cellular panning approach assisted by next-generation sequencing (NGS). Seven new FcγRI-specific antibody sequences were selected with this methodology, which were produced as Fc-silent antibodies showing FcγRI-restricted specificity.

## 1. Introduction

Antibodies activate immune cells via the constant fragment crystallizable (Fc) part by binding Fc receptors that are predominantly expressed on cells of the innate immune system. Human Fc γ receptors (FcγRs) bind the Fc part of IgG antibodies and can be divided into four activating receptors (FcγRI, FcγRIIa, FcγRIIc, FcγRIIIa), one inhibitory receptor (FcγRIIb) and one non-signaling receptor (FcγRIIIb). Furthermore, FcγRs can be distinguished by their affinity for monomeric IgG, with FcγRI being the only high-affinity FcγR in humans [1,2]. Antibodies that can specifically bind FcγRI via their fragment for antigen binding (Fab) are useful tools for FcγRI detection in diagnostics and research. Furthermore, FcγRI-specific antibodies can be of interest for clinical applications as components in bispecific antibodies to engage immune cells or as blocking antibodies for overactive immune cells in autoimmunity and chronic inflammatory diseases like rheumatoid arthritis [3,4].

Several monoclonal antibodies against FcγRs have successfully been generated by hybridoma technology (e.g., 3G8 for FcγRIII or IV.3 for FcγRII) [5,6]. Here, antibody-producing B cells from immunized mice are fused with mouse myeloma cells and separated to generate immortalized single clones that constantly produce monoclonal antibodies [7]. Antibodies in the supernatant of hybridoma cells can be screened for specific binding to the receptor of interest expressed on the cell surface. However, this approach is limited when used for human FcγRI since monomeric mouse IgG2a, IgG2b, and IgG3 will bind to human FcγRI via the Fc part with high affinity [8,9]. Hybridoma clones that produce monoclonal antibodies of these isotypes will lead to false-positive signals on FcγRI-positive cells, even if there is no Fab-mediated FcγRI binding. Incubating FcγRI with an excess of human IgG or Fc in order to block interaction with monomeric mouse IgG may favor the selection of Fab-mediated antibody binding. Although FcγRI-specific hybridoma clones have been isolated in the presence of hIgG (10.1 and m22, both mIgG1), limitations remain since bound Fc can bias the selection process by blocking potential epitopes for new antibody candidates [10,11]. These problems can be overcome by phage display because antibodies will be displayed on phages as antibody fragments without an Fc part and can therefore be screened for FcγRI specificity without any compromising Fc-FcγRI interaction.

We generated a single-chain fragment variable (scFv) antibody library from a mouse immunized with FcγRI-transduced cells. To screen for FcγRI-specific antibodies, phage display with a cellular screening approach on mouse cells transduced to express human FcγRI was performed, and individual scFv antibody candidates were analyzed for FcγRI-specific binding. A panel of seven chimeric, Fc-silent antibodies was generated, which specifically bind to human FcγRI.

## 2. Materials and Methods

### 2.1. Culture of Eukaryotic Cells

Ba/F3 and IIa1.6 (American Type Culture Collection) were cultured in RPMI 1640 medium (RPMI 1640 Glutamax, Thermo Fisher Scientific, Waltham, MA, USA) supplemented with 10% fetal calf serum (FCS, Thermo Fisher Scientific, Waltham, MA, USA), 100 U/mL penicillin/streptomycin (Pen/Strep, Thermo Fisher Scientific, Waltham, MA, USA). Medium for Ba/F3 cells was supplemented with 0.2 ng/mL murine Interleukin-3 (improved sequence) (mIL-3 IS; Miltenyi Biotec, Bergisch Gladbach, Germany). Ba/F3 cells were transduced with human FcγRI (GenBank accession no. L03418) using amphotropic viral particles made in HEK293T cells. The retroviral vector pMX containing human FcγRI has been previously described [12]. Stable Ba/F3-FcγRI cell populations were selected and maintained by puromycin (5 µg/mL). For the FcγRI-specificity analysis, IIa1.6 cells were transduced with FcγRIIa (H) and FcγRIIb, and Ba/F3 cells were transduced with FcγRIIIa (158F) and FcγRIIIb. Expression of FcγRII and FcγRIII was controlled by cytometry using the fluorochrome-conjugated antibodies 3G8 and FL18.26, respectively (all BD, Franklin Lakes, NJ, USA).

### 2.2. Generation of F(ab’)_2_ from IgG

One milligram of antibody (in-house-produced FcγRI hybridoma clones 1E2 and 1E3 and antibodies 197 and 10.1) was digested with pepsin using the Pierce F(ab’)_2_ preparation kit (Thermo Fisher Scientific, Waltham, MA, USA), following the manufacturer’s protocol. To determine purity, F(ab’)_2_ preparations were subjected to sodium dodecyl sulfate–polyacrylamide gel electrophoresis (SDS-PAGE) (reducing condition, 4–20% gradient gel; Bio-Rad, Hercules, CA, USA) and stained using InstantBlue Ultrafast Protein Stain (Sigma-Aldrich, Burlington, MA, USA).

### 2.3. Flow Cytometric Analyses with Antibodies and F(ab’)_2_

For flow cytometric analysis, 1 × 10^5^ cells/well were seeded to a 96-well plate and washed with PBS. Next, 10 µg/mL of full antibody or purified F(ab’)_2_ was added and incubated at 4 °C for 1 h. Cells were washed twice with PBS, and 5 μg/mL APC-labeled goat F(ab’)_2_ anti-mouse IgG (H + L, Thermo Fisher Scientific, Waltham, Massachusetts, USA) was added for 1 h at 4 °C. In the case of FITC-labeled antibodies, cells were seeded at 1 × 10^6^ cells per well. FITC-labeled antibodies were added at 10 µg/mL and incubated for 45 min at 4°. After the last washing steps, cells were fixated with 1% paraformaldehyde for 15 min at 4 °C and measured on FACSCanto II (BD, Franklin Lakes, NJ, USA). Analysis was performed with the accompanying software (FACS DIVA 9.0.1).

### 2.4. Generation of Murine scFv Antibody Library from Spleen

Animal experiments were approved by the local Animal Ethical Committee (WP-104603-2). C57BL/6 mice were immunized intravenously (i.v.) with a mixture of UMi-FcγRI (immunization) and UMe-FcγRI (enhancement) cells (1.5 × 10^5^ cells, ratio 1:1). Mice were boosted 4 times by i.v. injection of 1.5 × 10^5^ UMe-FcγRI cells. By using a combination of UMi and UMe cells, strong antibody titers can be induced. These cells are developed by and are proprietary to the UMAB facility of UMC Utrecht, the Netherlands, and were maintained in RMPI supplemented with 10% FCS and 100 U/mL Pen/Strep (all Thermo Fisher Scientific, Waltham, MA, USA). Blood samples were collected and serum antibody titers were analyzed by flow cytometry. Ba/F3-FcγRI cells were blocked for 30 min with 5 µg/mL hIgG Fc part, after which pre-diluted serum was added for 1 h. After washing with PBS, goat F(ab’)_2_ anti-mouse IgG-APC was added for 45 min, fixated with 1% paraformaldehyde, and measured on FACSCanto II (BD, Franklin Lakes, NJ, USA). The mouse with the highest antibody titers for FcγRI-positive cells was chosen. The spleen was harvested 4 days after the last boost (day 91) and stored at −80 °C following snap freezing. Total RNA from the spleen was isolated using the RNeasy Mini kit (Qiagen, Hilden, Germany), according to the manufacturer’s protocol. A murine scFv antibody library was generated from RNA as previously described [13]. Briefly, cDNA was synthesized from total RNA using oligo(dT)_15_ primers and reverse transcriptase. Mouse variable regions of the heavy and light chains (VHs and VLs) were amplified by PCR, assembled to scFvs by cloning, and inserted in the pAK100 phagemid [14].

### 2.5. Phage Display Using Cellular Panning

Phages were prepared from *E. coli* and titrated as described [13]. For the depletion of unspecific Ba/F3 binders, 2 × 10^7^ Ba/F3 cells were incubated with 10^11^–10^12^ phages in 2 mL PBS supplemented with 4% bovine serum albumin (BSA) for 1 h on a roller incubator at room temperature. Cells were separated by centrifugation (500× *g*, 5 min), and the supernatant was added to 2 × 10^6^ Ba/F3-FcγRI (previously blocked in PBS supplemented with 4% BSA for 30 min at room temperature). After 1 h incubation on a roller incubator at room temperature, the cells were washed 10 times with PBS supplemented with 2% BSA and 2 times using PBS (300× *g*, 4 min). For elution, cells were incubated in 1.5 mL 50 mM HCl solution for 10 min at room temperature and neutralized with 0.5 mL 1 M TRIS/HCl buffer (pH 7.5). Cells were separated by centrifugation (18,000× *g*, 10 min) and *E. coli* were infected with eluted phages as described [13]. Pooled *E. coli* were used to prepare phages, and a second cellular panning step was performed as described above without a depletion step with Ba/F3 cells.

### 2.6. Flow Cytometric Analyses with Polyclonal Phage Antibodies

First, 5 × 10^5^ cells were incubated with 10^11^ phages in blocking buffer (PBS supplemented with 1% BSA and 0.1% NaN_3_) for 1 h at 4 °C and washed three times with blocking buffer. Phages displaying an scFv targeting human CD7 were used as a control [15]. For the detection of phages, cells were first incubated for 1 h at 4 °C with recombinant human anti-myc Fab (40 µg/mL, Abnova, Taipeh, Taiwan) and washed three times with blocking buffer. Afterwards, cells were incubated with polyclonal anti-human kappa light chain FITC-labeled F(ab’)_2_ (SouthernBiotech, Birmingham, AL, USA) for 1 h at 4 °C, washed three times with blocking buffer and measured on a Navios flow cytometer (Beckman Coulter, Brea, CA, USA). Analysis was performed with Kaluza software 1.3 (Beckman Coulter, Brea, CA, USA).

### 2.7. Whole Cell ELISA with Monoclonal Phage Antibodies

Monoclonal phage antibodies were prepared from individual *E. coli* colonies as described [13]. For whole-cell ELISA, 96-well plates were blocked with PBS supplemented with 4% BSA. Then, 1 × 10^6^ Ba/F3 or Ba/F3-FcγRI cells per well were incubated for 30 min on ice with PBS supplemented with 4% BSA. A 100 µL volume of bacterial supernatant containing monoclonal phage antibodies was added to the cells and incubated for 1 h on ice. Plates were washed three times with cold PBS supplemented with 0.1% BSA (680× *g*, 5 min, 4 °C). For phage detection, cells were incubated with mouse anti-M13 HRP-conjugated IgG1 antibody (Creative Diagnostics, Shirley, NY, USA; diluted 1:2000 in PBS supplemented with 4% BSA) for 1 h on ice. After washing, 100 µL ABTS solution (Roche, Basel, Switzerland) was added per well, and absorbance was measured at 405 nm with a Sunrise absorbance microplate reader (reference wavelength 492 nm; Tecan, Männedorf, Switzerland).

### 2.8. Sequencing Analyses

For Sanger sequencing, phagemids were prepared from a bacterial culture of separated *E. coli* colonies (16 h in 5 mL 2xYT medium supplemented with 1% glucose, 30 μg/mL chloramphenicol, and 10 μg/mL tetracycline; all Carl Roth, Karlsruhe, Germany) using NucleoSpin Mini kit (Macherey-Nagel, Düren, Germany). Sanger sequencing of the VH (Primer: CGTATGTTGTGTGGAATTGTGAGCGG) and VL (Primer: CATAGCCCCCTTATTAGCGTTTGCC) of phagemids was performed using standard procedures. Nucleotide sequences were translated into amino acid sequences and aligned by ClustalW using Neighbor Joining to describe similarities in dendrograms. Since the seven N-terminal and seven C-terminal amino acids are covered by the degenerate primers used for V region amplification, mutations in these positions were excluded from analysis.

For NGS, phagemids from pooled bacterial colonies of the initial scFv antibody library the antibody library after the first panning and the antibody library after the second panning were prepared using the NucleoBond Xtra Maxi kit (Machery-Nagel, Düren, Germany). Preparation of samples and NGS was performed as previously described [13]. Briefly, VHs were amplified by PCR to add adapters and indexes. Products were sequenced using the Illumina MiSeq system (MiSeq Reagent Kit v3, 2 × 300 nt reads; Illumina, San Diego, CA, USA). Clonotypes of IGHV-IGHD-IGHJ rearrangements (hereon “VH”) were identified by ARResT/Interrogate [arrest.tools/interrogate] and frequencies were calculated by in-house Python scripts [13,16].

### 2.9. Generation of Chimeric FcγRI-Specific Antibodies

Selected scFvs were converted into IgG1 antibodies. Therefore, overlap extension (OE)-PCR was performed to introduce a *Hind*II restriction site, Kozak sequence, HAVT20 signal peptide, splice donor sites (‘GTGAGT’ for VH and ‘GTAAGTAGTCTTCTCAA’ for VL), and *Nhe*I and *Not*I restriction sites. After OE-PCR, the VL and VH fragments were inserted into an Fc-silent human IgG1 backbone containing the mutations L234A, L235A, and P329G [17]. Selected clones were produced by transfection in ExpiCHO-S cells (Thermo Fisher Scientific, Waltham, MA, USA), following the manufacturer’s protocol. The supernatant was harvested and filtered using 0.4 μm and 0.22 μm membranes (Express PLUS membrane filters; Merck Millipore, Burlington, MA, USA). Antibodies were purified using a HiTrap rProtein A FF column (GE Healthcare, Chicago, IL, USA) attached to the ÄKTA Start (GE Healthcare, Chicago, IL, USA) fast protein liquid chromatography system according to the manufacturer’s protocol. Bound antibodies were eluted with 0.1 M sodium acetate pH 2.5 (Sigma-Aldrich, Burlington, MA, USA) and neutralized with 1 M TRIS/HCl (pH 8.8; Roche, Basel, Switzerland). Antibody fractions were concentrated using centrifugal concentrators (Vivaspin 20, 100 kDa MWCO, Cytiva, Marlborough, MA, USA) and dialyzed overnight in PBS using the Slide-A-Lyzer Dialysis Cassette 10K 3 mL or 12 mL (Thermo Fisher Scientific, Waltham, MA, USA). Concentration was measured by UV absorbance (NanoDrop; Thermo Fisher Scientific, Waltham, MA, USA) and high purity was verified by SDS-PAGE following InstantBlue (Sigma-Aldrich, Burlington, MA, USA) staining.

### 2.10. Generation, Stimulation, and Labeling of Polymorphonuclear Cells

Human polymorphonuclear cells (PMNs) were isolated from healthy donor blood by Ficoll-density gradient centrifugation. Blood was obtained via our in-house, volunteer-based blood donation system. The volunteers, all employees or students at the UMC Utrecht, had to sign an informed consent form for their blood to be used for research purposes. The system was approved by the Board of Directors of the UMC Utrecht after review by the independent Review Committee Biobanks. Red blood cell lysis buffer (RBC lysis 10× BioLegend, San Diego, CA, USA) was used to lyse the erythrocytes in the PMN fraction. PMNs were stimulated for 16 h with 100 U/mL interferon-γ (IFN-γ) and granulocyte colony-stimulating factor (G-CSF) in FCS-supplemented RPMI medium to induce FcγRI expression. For binding analysis, IFN-γ-stimulated PMNs were seeded at 1 × 10^6^ cells per well. FITC-labeled antibodies were added at 10 µg/mL and incubated for 30 min at 37 °C. After washing, cells were fixated with 1% paraformaldehyde for 15 min at 4 °C and measured on FACSCanto II (BD, Franklin Lakes, NJ, USA). Analysis was performed with the accompanying software (FACS DIVA 9.0.1).

## 3. Results

### 3.1. Isolation of FcγRI-Specific Antibodies by Hybridoma Technology Is Biased by Fc-Mediated Binding

**Figure 1:**
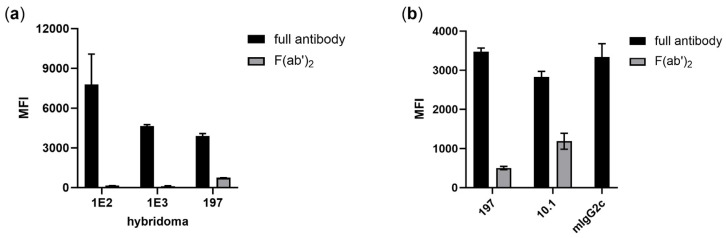
Antibodies from hybridoma clones selected for FcγRI specificity lack or show reduced binding to FcγRI after elimination of the Fc part. (**a**) F(ab’)_2_ were prepared from antibodies of hybridoma clones (in-house-produced 1E2 and 1E3, mIgG2c) and 197 (mIgG2a) and show missing or strongly reduced binding to Ba/F3-FcγRI after elimination of the Fc part. (**b**) For comparison, commercially available FcγRI antibodies 197 (mIgG2a) and 10.1 (mIgG1) also show a reduced signal but remain able to bind to Ba/F3-FcγRI after elimination of the Fc part. The isotype of the in-house-produced hybridoma (mIgG2c) mediates Fc-restricted binding to human FcγRI. Data represent mean values of two to five experiments +/− SEM. Cells were stained with 10 µg/mL antibodies and APC-labeled goat F(ab’)_2_ anti-mouse IgG (H + L). MFI: mean fluorescence intensity.

Previously, we aimed to produce FcγRI-specific antibodies by immunization of mice following hybridoma technology. FcγRI-specific binding of antibodies from hybridoma supernatants was tested by using FcγRI-transduced Ba/F3 cells. Although cells were pre-incubated with hIgG to block FcγRI and reduce the risk of Fc-mediated binding during selection, a bias towards Fc-FcγRI binding can be observed: antibodies from two hybridoma clones (1E2 and 1E3) produced in-house show FcγRI-restricted binding properties as purified IgG but lose binding as F(ab’)_2_ (Figure 1a). Both antibodies are of the mIgG2c isotype, which shows Fc-mediated binding to human FcγRI, and were probably selected due to the Fc-FcγRI interaction. A similar effect can be observed for the published mIgG2a FcγRI antibody produced from the hybridoma clone 197, although a strongly reduced signal remains (Figure 1a,b) [18]. For comparison, though the signal of the FcγRI antibody 10.1 is reduced, a clear FcγRI-restricted signal remains after elimination of its mIgG1 Fc part that is supposed to have a neglectable affinity to human FcγRI [11]. In summary, these results demonstrate that using hybridoma technology is challenging for the generation of antibodies against the human high-affinity receptor FcγRI.

### 3.2. Isolation of FcγRI-Specific Antibodies by Phage Display Using Cellular Panning Obviating Fc-FcγRI Interactions

**Figure 2:**
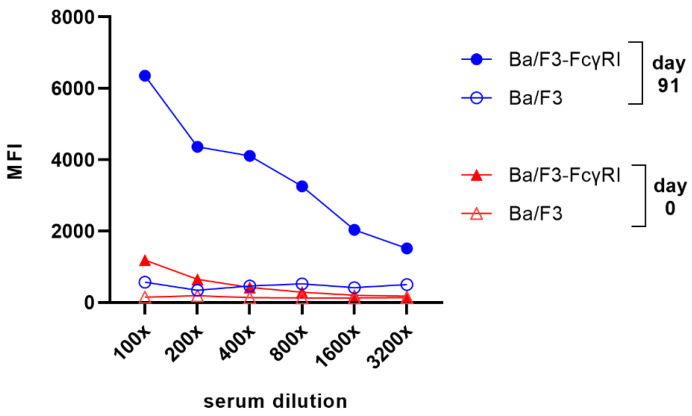
Binding of serum antibodies to FcγRI-transduced mouse cells after cellular immunization using a flow cytometry-based screening method. Ba/F3 and Ba/F3-FcγRI were first blocked with hIgG Fc, to reduce the Fc-mediated binding of serum antibodies. Serum was stepwise diluted with the dilution factor indicated. Sera from day 0 (before immunization) and day 91 (final bleed) were tested. Antibody binding was determined with a goat F(ab’)_2_ anti-mouse IgG-APC antibody. Screening data of the mouse with the highest antibody titers for FcγRI-positive cells are shown. MFI: mean fluorescence intensity.

Mice were immunized with FcγRI-transduced cells and blood was taken to assess whether the immunization was successful and whether FcγRI-specific antibodies were present in the polyclonal mouse serum. Therefore, Ba/F3 and Ba/F3-FcγRI (blocked with an excess of hIgG Fc) were incubated with diluted serum and binding of mouse antibodies was measured by flow cytometry. Before immunization (day 0), only a small increase in mean fluorescence intensity (MFI) comparing Ba/F3 and Ba/F3-FcγRI can be observed at low dilution factors (Figure 2, red triangles). After the final bleed (day 91), a higher signal for Ba/F3-FcγRI can be observed compared to the serum sample before immunization (Figure 2, blue circles vs. red triangles).

For the generation of the scFv antibody library, total RNA was prepared from the spleen of the mouse with the highest antibody titers for FcγRI-positive cells and used as a template for cDNA synthesis. VH and VL regions were amplified from cDNA by PCR and assembled to scFvs before insertion into the pAK100 phagemid. The final antibody library had a size of 6 × 10^6^ colony-forming units (CFU) and was used for phage display. A cellular panning approach was performed including a depletion step with non-transduced Ba/F3 cells and selection steps with FcγRI-transduced Ba/F3 cells. After the first panning, an output of 19,200 CFU eluted phages was measured and an output of 617,000 CFU was observed after the second panning. Therefore, a clear enrichment by factor 54 was observed (Table 1).

**Figure 3:**
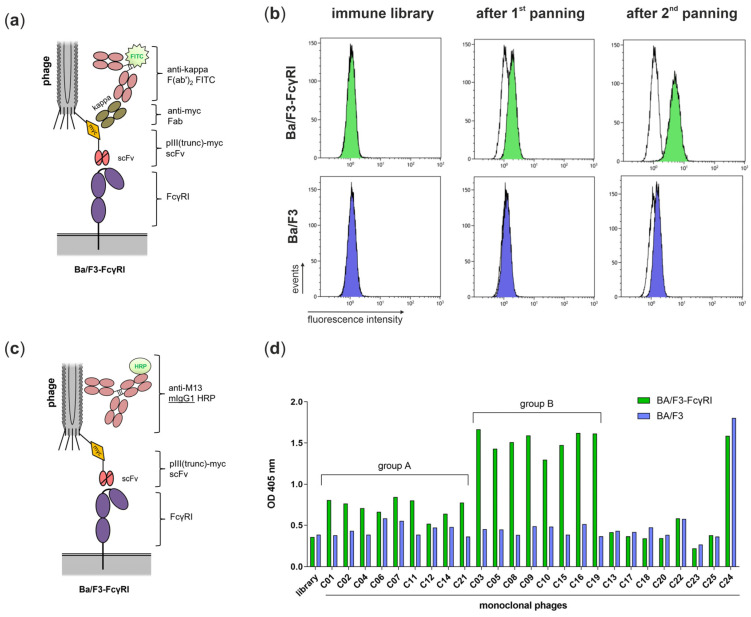
FcγRI-specific scFvs were enriched and selected from an antibody library by phage display. (**a**) Schematic overview of “Fc-free” detection of bound phages in flow cytometric analyses using anti-myc Fab and polyclonal anti-kappa light chain FITC-conjugated F(ab’)_2_. (**b**) Flow cytometric analysis of phage antibodies of the initial library, after the 1st panning and after the 2nd panning. Ba/F3-FcγRI (green) and Ba/F3 (blue) cells were incubated with polyclonal phages and fluorescence was measured in comparison to a control phage (white). (**c**) Schematic overview of initial whole-cell ELISA experiments using anti-M13 HRP-conjugated mouse IgG1 for the detection of cell-bound phages. (**d**) A total of 1 × 10^6^ Ba/F3-FcγRI (green) or Ba/F3 (blue) cells were incubated with monoclonal phage antibodies from 25 single bacterial colonies (C01–C25), and bound phages were detected with anti-M13 HRP-conjugated mouse IgG1. For comparison, polyclonal phage antibodies of the initial library were added to the analysis. Candidates binding with higher intensity to FcγRI-transfected cells than to non-transfected Ba/F3 cells can be grouped according to their intensities in group A (OD 0.5–1) and group B (OD 1–2).

For the screening of candidates, a detection antibody that favored the Fc-FcγRI interaction (e.g., mIgG2a or mIgG2c) had to be avoided. Therefore, an “Fc-free” detection system for bound phages in flow cytometric analyses was applied. The incorporated myc tag between the truncated phage coat protein pIII and scFv was used for detection with recombinant anti-myc Fab followed by polyclonal FITC-conjugated anti-kappa F(ab’)_2_ as a secondary antibody (Figure 3a). The polyclonal phages from the initial antibody library show no binding to FcγRI-transduced Ba/F3 cells, but an increased signal after the first and second panning can be observed compared to control phages (Figure 3b, green). For non-transduced Ba/F3 cells, no binding was measured for the initial library or after the first panning, but it was slightly detected after the second panning (Figure 3b, blue). However, the MFI for FcγRI-transduced Ba/F3 cells was stronger than for the non-transduced Ba/F3 cells (5.41 vs. 1.65), demonstrating FcγRI-specific candidates. After the second panning, *E. coli* colonies were separated in order to prepare monoclonal phages representing individual antibodies. Candidates were tested in whole-cell ELISA with FcγRI-transduced and non-transduced Ba/F3 cells. To avoid an interaction of detection antibodies with Fc receptors, an HRP-conjugated mouse IgG1 anti-M13 phage antibody was used for detection in this setting (Figure 3c). In contrast to mIgG2a/c and mIgG3, mIgG1 does not bind to human FcγRI [8,9]. Twenty-five monoclonal phage antibodies (C01–C25) were prepared, analyzed by ELISA, and grouped by signal intensity and FcγRI specificity. In total, 68% of the candidates have a higher signal for FcγRI-transduced Ba/F3 cells than for non-transduced Ba/F3 cells and can be rated as FcγRI-specific (Figure 3d). From those, 53% show a weak signal (OD 0.5–1, group A) and 47% a strong signal (OD 1–2, group B). However, 32% of the 25 monoclonal phages tested show no FcγRI-specific binding and one of them (C24) a strong signal on Ba/F3 cells, probably binding a different antigen than FcγRI (Figure 3d).

### 3.3. The Panel of FcγRI-Specific scFvs Can Be Grouped in Two VH Clonotypes

**Figure 4:**
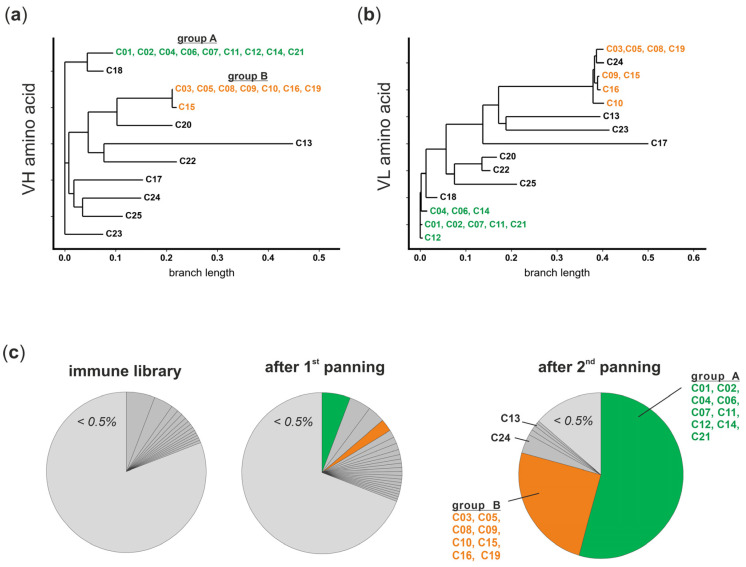
Enrichment of FcγRI-specific antibodies was monitored by NGS. (**a**) VH and (**b**) VL amino acid sequences were aligned and are shown as dendrograms. Eight candidates that show the same VH are combined with three different VLs and can be assigned to group A (green). Seven candidates that show the same VH are combined with four different VLs and can be assigned to group B (orange). The VH of C15 differs in one amino acid from the candidates of group B. (**c**) VHs of the initial immune library the library after the 1st panning and the library after the 2nd panning were sequenced by NGS, the clonotypes were determined by ARResT/Interrogate, and the frequencies (% of VH sequences) in each sample were calculated. Clonotypes with a low frequency (<0.5%, light gray) are summed up. After the 2nd panning, 54% and 25% of all VH sequences share the same clonotypes of groups A (green) and B (orange), respectively.

To determine the genetic background, the scFvs of all 25 candidates were Sanger sequenced. Amino acid sequences of VHs and corresponding VLs were aligned and similarities described as dendrograms (Figure 4a,b). All nine scFv phage antibodies of group A share the same VH amino acid sequence and three closely related VL sequences are distinguished only by two amino acid positions (Figure 4a,b, indicated in green). In group B, seven of eight candidates had the same VH amino acid sequence, while an additional candidate differed from those in only one amino acid (C15). Again, four closely related VLs were identified, distinguishing in five amino acid positions (Figure 4a,b, indicated in orange). Taken together, scFvs that differ in VH and/or VL amino acid sequences were identified and can be grouped in two clusters representing the two groups identified according to their FcγRI-binding intensity. Amino acid sequences of remaining candidates without FcγRI-specific signals were scattered cross alignments without showing tendencies to cluster (Figure 4a,b, indicated in black).

To get a closer look into the antibody library composition, deep sequencing of the VH in the initial library and the libraries after the first and second panning were performed. Clonal distribution (IGHV-IGHD-IGHJ rearrangements) in the libraries and the underlying B-cell population were analyzed. In the initial library, high diversity was observed since 81% of sequences were assigned to clonotypes with frequencies (% of sequenced VH) lower than 0.5%. After the first panning, this parameter decreased (69%), and after the second panning, only 14% of sequences were represented by clonotypes with frequencies lower than <0.5% (Figure 4c, light grey). In contrast, two dominant clonotypes (54% and 25%) appeared after the second panning, which can be assigned to the candidates of group A and group B identified by Sanger sequencing and in initial whole-cell ELISA (Figure 3d and Figure 4a,b). Both clonotypes were also found after the first panning (5.8% and 2.5%, respectively) and had a low frequency (<0.5%) in the initial library, proving their strong enrichment by panning on FcγRI-transduced Ba/F3 cells. Furthermore, the third-most-frequent clonotype (3.8%) and the sixth-most-frequent clonotype (0.7%) can be assigned to C24 and C13, respectively, after the second panning (Figure 4c). Both showed an unspecific signal or no signal in initial whole-cell ELISA.

### 3.4. Chimeric Fc-Silent Antibodies Show FcγRI-Specific Binding

**Figure 5:**
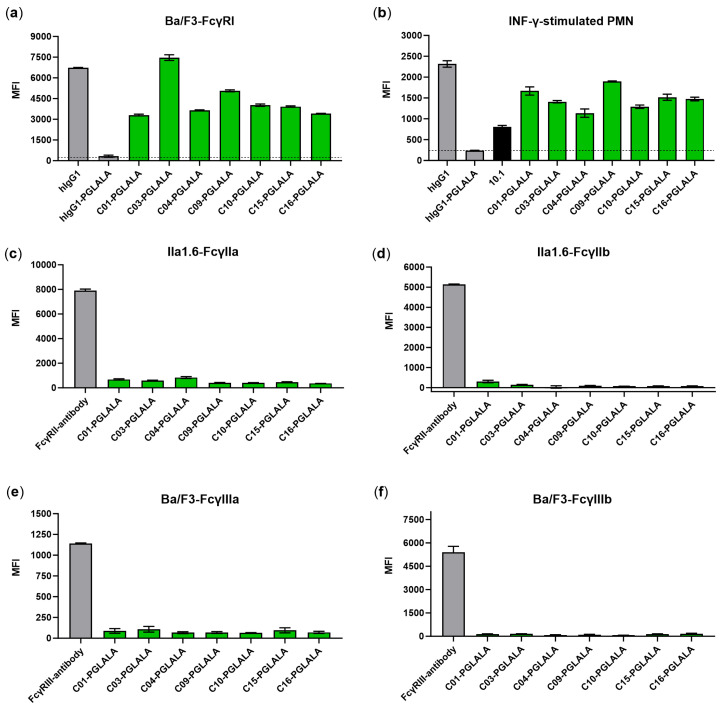
Selected scFvs show Fab-mediated and specific binding to FcγRI after conversion in Fc-silent IgG1. Antibody candidates, produced as chimeric IgG1 with LALAPG mutations, show Fab-mediated binding (green) to (**a**) Ba/F3-FcγRI cells and (**b**) IFN-γ-stimulated PMNs in cytometric analysis. As proven by control antibodies, inserting LALAPG mutations into human IgG1 (IgG1-LALAPG, dotted lines) silences Fc-mediated binding to human FcγRI compared to human wildtype IgG1 (grey). The FcγRI-specific antibody 10.1 (mIgG1) was used as a positive control (black). To exclude cross-reactivity with other human FcγRs, binding to (**c**,**d**) FcγRIIa/b-transduced IIa1.6-cells and (**e**,**f**) FcγRIIIa/b-transduced Ba/F3 cells was measured by flow cytometry, showing FcγRI-specific binding of the generated antibodies (green) in comparison to FcγRIIa/b- and FcγRIIIa/b-specific antibodies (grey). Data represent mean values of two to three experiments +/− SEM. Cells were stained with 10 µg/mL of FITC-labeled antibodies. MFI: mean fluorescence intensity.

The unique candidates C01 and C04 from group A and unique candidates C03, C09, C10, C15, and C16 from group B had different amino acid sequences in VH and/or VL and were chosen for further analysis. All candidates were converted from scFv to mouse/human chimeric IgG1 antibodies carrying Fc-silencing mutations (L234A, L235A, P329G; LALAPG). Compared to the human IgG1 with wildtype Fc, control IgG1-LALAPG showed strongly reduced Fc-mediated binding to Ba/F3-FcγRI and FcγRI-positive IFN-γ-stimulated PMNs (Figure 5a,b, grey). In contrast, clear signals can be observed for C01, C04, C03, C09, C10, C15, and C16 IgG1 antibodies with LALAPG mutations, indicating Fab-mediated FcγRI binding for FcγRI-transduced Ba/F3 and PMNs that become FcγRI-positive after stimulation with IFN-γ (Figure 5a,b, green).

To further analyze if the new FcγRI-specific antibodies showed cross-reactivity with the human receptors FcγRIIa/b and FcγRIIIa/b, flow cytometric analysis was performed with FcγRIIa/b-transduced IIa1.6 cells and FcγRIIIa/b-transduced Ba/F3. As shown in Figure 5c–f, none of the antibodies from the panel of new FcγRI-specific antibodies bound to FcγRs other than FcγRI.

## 4. Discussion

A panel of FcγRI-specific antibodies was isolated from an immune antibody library generated from mice immunized with a unique cellular immunization protocol and screened by phage display using a cellular panning approach. FcγRI-specific scFvs were enriched and individual candidates were characterized, leading to seven chimeric, Fc-silent IgG antibodies with FcγRI-specific binding.

Hybridoma technology is an established method in immunology and is successfully used for the generation of monoclonal antibodies. However, problems arise if antibodies from the supernatant of hybridoma clones interact Fab-independently with target proteins used for screening like the human high-affinity FcγRI. This could be demonstrated for the previously described hybridoma clone 197 (mIgG2a), which clearly loses binding to FcγRI after elimination of its mIgG2a Fc part. Although FcγRI-specific hybridoma clones of the mouse isotype IgG1 were isolated in the presence of hIgG (10.1 and m22), the limitations remain, including blocking of potential epitopes [10,11]. In our approach, both cellular immunization and cellular panning strategies were combined using FcγRI-transduced cells. Under these conditions, the FcγRI will be displayed on the eucaryotic cell in its naïve, membrane-integrated form, including post-translational modifications like glycosylation [19]. Furthermore, the presence of human IgG or Fc parts was avoided both during the selection and detection of antibody fragments. Candidates were successfully enriched after the second panning, compared to established panning protocols requiring up to four rounds of panning [20]. Furthermore, the panning process was monitored in detail by NGS of the VH as previously described [13]; hence, the composition of the initial library and the enrichment of individual antibody fragments could be investigated. FcγRI-binding scFv phage antibodies turned out to group into two clusters showing similar binding properties and VH clonotypes, therefore probably originating from two different naïve B cells which further diverged in sequences during maturation. Assuming that the initial antibody library reflects the composition of B-cell clones in the spleen after immunization, B cells producing FcγRI-specific antibodies had a low frequency in the spleen and probably would not have been identified by traditional hybridoma technology without extensive screening. One explanation for the low abundance of FcγRI binders could be the similarity between the human and mouse FcγRI (72.43% identity for the amino acid sequence of extracellular part; UniProt P12314 vs. P26151), which limits the access of epitopes due to self-tolerance. This may be potentially overcome by switching to antibody libraries that are not generated from an immunized mouse (e.g., synthetic or naïve antibody libraries), although antibodies from these sources do not undergo affinity maturation [21,22,23]. Our screening approach could be adapted for these types of antibody libraries.

## 5. Conclusions

For the identification of FcγRI-specific antibodies, an adapted phage display approach was applied including the generation of a mouse scFv immune antibody library after immunization with FcγRI-transduced cells and a cellular panning approach. Seven chimeric, Fc-silent FcγRI-specific antibodies that originated from two different B-cell clones were generated and will be further characterized regarding affinity, blocking of Fc-FcγRI interaction, and their epitopes to determine their potential in research or clinical usage.

## 6. Patents

The sequences of the identified antibodies have been described in related patent applications.

## Figures and Tables

**Table 1:** Enrichment of phages by cellular panning.

	Input Phages	Output Phages	Ratio	Enrichment
**after 1st panning**	10^12^ CFU	19,200 CFU	1.92 × 10^−8^	-
**after 2nd panning**	6 × 10^11^ CFU	617,600 CFU	1.03 × 10^−6^	54

CFU = colony-forming units; ratio = output phages/input phages; enrichment = 2nd ratio/1st ratio.

## Data Availability

The datasets presented in this article are available on request; please contact M.P. and J.H.W.L.
